# Expanding the Role of Heparin Derivatives in Oncology: From Anticoagulation to Antitumor Activity

**DOI:** 10.3390/ph18030396

**Published:** 2025-03-12

**Authors:** Jasmine Holail, Hatouf Husni Sukkarieh, Ahmad Aljada

**Affiliations:** 1 Department of Biochemistry and Molecular Medicine, College of Medicine, Alfaisal University, Riyadh 11533, Saudi Arabia; jholail@alfaisal.edu; 2 Department of Pharmacology, College of Medicine, Alfaisal University, Riyadh 11533, Saudi Arabia; hsukkarieh@alfaisal.edu

**Keywords:** heparin derivatives, low-molecular-weight heparins (LMWHs), non-anticoagulant heparins (NAC heparins), cancer therapy, antitumor activity, heparanase inhibition, angiogenesis inhibition, metastasis prevention

## Abstract

Current research demonstrates the expanding therapeutic potential of heparin derivatives in oncology, extending beyond traditional anticoagulation mechanisms. This systematic analysis examines the structural characteristics, molecular mechanisms, and therapeutic applications of heparin-based compounds in malignancy treatment. The essential antithrombin binding pentasaccharide sequence has enabled development of specialized molecular variants, particularly fractionated heparins and their non-anticoagulant counterparts. These agents exert antineoplastic effects via multiple pathways, particularly through modulation of heparanase enzymatic activity and specific protein–glycosaminoglycan interactions. Evidence from pivotal clinical trials (FRAGMATIC, MAGNOLIA, GASTRANOX) confirms efficacy in managing cancer-associated thrombosis while indicating potential enhancement of chemotherapeutic outcomes. The preparation methods utilize enzymatic cleavage reactions and selective chemical derivatization to generate structurally modified heparins exhibiting unique molecular characteristics and biological activities. Analysis of the glycosaminoglycan analog dociparstat sodium reveals significant activity in myeloid malignancies, mediated by specific interference with CXCL12/CXCR4 signaling cascades. Significant challenges remain in manufacturing scale-up, analytical validation, and long-term safety assessment. Future studies must address dose optimization, combination strategies, and controlled clinical trials to determine the full therapeutic potential of these compounds in clinical oncology.

## 1. Introduction

The isolation of heparin from canine liver cells in 1916 fundamentally changed the landscape of anticoagulation therapy. Biochemical studies during the 1920s characterized this compound’s distinctive structure, establishing it among the class of sulfated glycosaminoglycans. Laboratory investigations throughout the subsequent decades demonstrated remarkable diversity in both molecular composition and therapeutic potential [1,2,3].

The structural elements of heparin comprise a spectrum of molecular masses, predominantly ranging from 3000 to 30,000 daltons. Critical factors in biological activity include precise sulfation patterns, particularly within specific oligosaccharide sequences that determine anticoagulant potency [1,2,3]. Identification of the pentasaccharide sequence governing antithrombin (AT) binding led to rational design of therapeutic derivatives, advancing clinical options beyond traditional unfractionated heparin.

Mechanistic studies in the mid-20th century revealed dual inhibition pathways targeting both thrombin and factor Xa through antithrombin-mediated mechanisms. These insights guided development of low-molecular-weight heparin (LMWH) preparations, which offered superior pharmacological profiles compared to their unfractionated predecessors. Strategic molecular modifications produced compounds with enhanced subcutaneous bioavailability and more predictable anticoagulant responses. The therapeutic versatility of heparin derivatives expanded significantly following these developments, enabling applications across diverse clinical contexts.

Contemporary research extends well beyond heparin’s established role in coagulation. Experimental and clinical evidence demonstrates effects on inflammation, viral inhibition, and tumor progression. The compound’s interaction with growth factors, adhesion molecules, and enzymatic pathways suggests therapeutic applications in oncology warrant particular attention. Advances in structural biology have elucidated key heparin–protein binding mechanisms, providing a foundation for developing targeted heparin-based therapies for cancer. Emerging evidence indicates that heparin and its derivatives exert antineoplastic effects through multiple pathways, including inhibition of heparanase activity, disruption of selectin-mediated cellular adhesion, and modulation of growth factor signaling.

The systematic literature analysis encompassed primary research articles indexed in PubMed (MEDLINE), Web of Science, and Scopus databases from 1990–2024, focusing on structural characterization, molecular mechanisms, and clinical applications. Primary search strings incorporated specific Boolean operators combining “heparin AND (structure OR mechanism)” with “cancer OR neoplasm” and “LMWH OR low molecular weight heparin”. Selection criteria prioritized mechanistic studies elucidating structure–function relationships, randomized controlled trials evaluating clinical efficacy, and meta-analyses assessing therapeutic outcomes in oncology applications. This comprehensive review examines the expanding therapeutic potential of heparin derivatives in oncology, extending beyond traditional anticoagulation mechanisms to include direct antineoplastic effects, heparanase inhibition, and modulation of tumor microenvironment interactions.

## 2. Structural and Functional Properties of Heparin and Its Derivatives

Initial characterization of heparin in the 1920s, stemming from McLean’s 1916 discovery, marked the beginning of extensive research into this sulfated glycosaminoglycan [1]. Systematic laboratory studies conducted through the mid-20th century progressively elucidated its diverse biological activities, revealing capabilities beyond anticoagulation, including anti-inflammatory, antiviral, and antineoplastic properties [2,3]. Molecular investigations identified the critical pentasaccharide sequence responsible for AT binding and subsequent inhibition of thrombin and factor Xa. This structural insight led to development of low-molecular-weight heparin (LMWHs) derivatives, which demonstrate superior pharmacological profiles and reduced hemorrhagic risk in specific patient populations [1].

### 2.1. Molecular Structure and Anticoagulant Function

Laboratory studies examining anticoagulation mechanisms have identified structural elements governing heparin’s biological activity. Key molecular interactions occur between heparin and antithrombin III (AT III), which functions as a central regulatory protein in blood coagulation [4]. Within the heparin molecule, researchers identified a unique pentasaccharide motif that binds AT III with remarkable specificity, enhancing its capacity to inhibit both factor Xa and thrombin [5]. Structural investigations highlight the presence of eight sulfate groups arranged in a precise spatial configuration. These sulfate groups, particularly those positioned at the reducing-end disaccharide, exhibit synergistic effects in achieving optimal anticoagulant function [6].

**Figure 1:**
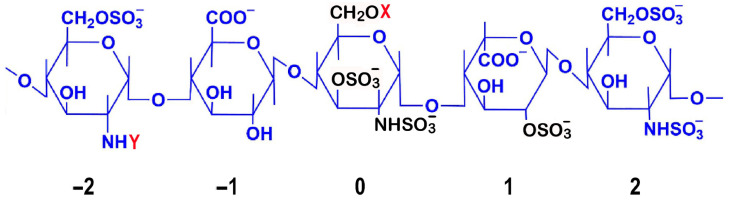
Structural and Functional Analysis of the Heparin AT III Pentasaccharide Binding Site. The central residue is numbered 0, while the non-reducing and reducing terminal residues are numbered −2 and 2, respectively. Critical sulfation points essential for AT III binding are highlighted in black: on the central glucosamine unit (position 0), the most critical 3-O-sulfation, N-sulfation (NHSO_3_^−^), and 6-O-sulfation; and on the iduronic acid at position 1, the 2-O-sulfation. Commonly observed structural variations are indicated, where X can be H or SO_3_^−^ and Y can be -COCH_3_ or SO_3_^−^. The AT III binding pentasaccharide sequence has specific structural requirements: the central unit (0) is most critical, requiring 3-O-sulfation, N-sulfation (NHSO_3_^−^), and a flexible 6-O position (CH_2_OX). The flanking glucosamine unit at position −2 can be either N-sulfated (NHSO_3_^−^) or N-acetylated (NHCOCH_3_), contributing to structural variability. 6-O-sulfation is only possible on glucosamine units (positions −2, 0, and 2), while hexuronic acid residues (−1 and +1) lack the necessary hydroxyl group for 6-O-sulfation but contain carboxyl groups (COO⁻) that contribute to the overall negative charge. The flanking units (−2, −1, 1, 2) provide key binding contacts: N-sulfate groups (NHSO_3_^−^) on glucosamine units −2 and 2, 6-O-sulfates on these glucosamine units to enhance binding affinity, and 2-O-sulfates on uronic acid units (−1 and 1) to maintain the correct conformation. Binding causes a conformational change in AT III, exposing its reactive center loop to more efficiently inhibit coagulation factors, especially factor Xa. This mechanism underlies heparin’s anticoagulant activity and enabled development of synthetic drugs like fondaparinux. Natural structural variation, with X being H or SO_3_^−^ and Y being -COCH_3_ or SO_3_^−^, affects AT III binding affinity and is important for structure–activity relationships and drug development. Anticoagulant potency is influenced by the level of sulfation, with conserved structural features essential for AT binding. These include the critical 3-O-sulfate group on the central glucosamine unit, carboxylate groups, the core pentasaccharide sequence, and the specific sulfation pattern at other positions [9,10,11].

Detailed mapping of sulfation patterns revealed position-specific roles: the 3-O-sulfate modification on the central glucosamine (position 0) emerges as crucial for AT III binding, while the 6-O-sulfation on the glucosamine at position −2 and 2-O-sulfation of iduronic acid at position 1 are essential for both factor IIa and Xa inhibition [7] (Figure 1). Notably, the iduronic acid residue maintains dynamic conformational states, alternating between chair and skew-boat arrangements to facilitate optimal AT III interactions [8].

Modern analytical platforms—X-ray crystallography, high-field NMR spectroscopy, and mass spectrometric techniques—have enabled precise mapping of heparin’s structural elements [12]. These complementary methods revealed complex details of the molecule’s spatial organization, with particular focus on the pentasaccharide domain responsible for AT III recognition. Structural biology studies demonstrate that sequences flanking this core pentasaccharide, while not directly involved in AT III binding, significantly influence heparin’s overall biological profile through protein–glycan interactions [13,14]. Integration of these structural insights drove development of next-generation anticoagulants including synthetic compounds like fondaparinux and idraparinux, as well as semi-synthetic LMWHs. These derivatives exhibit enhanced pharmacological properties, specifically increased factor Xa selectivity coupled with reduced adverse effects compared to unmodified heparin [15].

**Figure 2:**
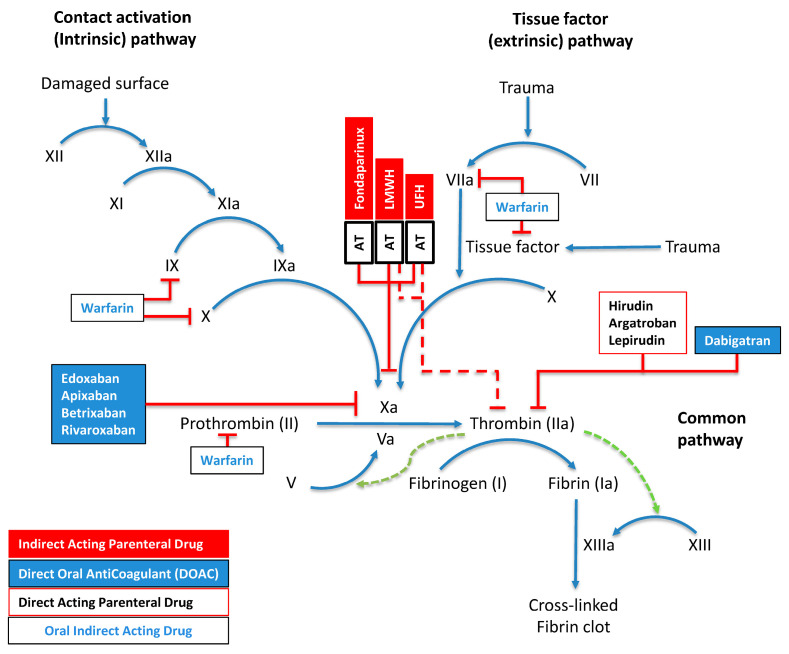
Molecular Mechanisms of Hemostasis and Anticoagulation. This schematic delineates the interconnected pathways of blood coagulation, comprising contact-activation-mediated (intrinsic) and tissue-factor-induced (extrinsic) cascades that converge to orchestrate thrombus formation. The diagram uses standardized nomenclature where activation sequences appear as blue directional arrows, regulatory feedback loops as green dashed vectors, and inhibitory mechanisms as red perpendicular terminators. Sequential activation by zymogens (Roman numerals) of their serine proteases (suffix “a”) drives the process. Four pharmacological anticoagulant classes are shown: parenteral indirect-acting agents (red boxes), direct oral anticoagulants (DOACs; blue boxes), parenteral direct-acting compounds (black-bordered white boxes), and indirect-acting oral therapeutics (blue text in white boxes). Multiple regulatory checkpoints exist through AT complexes, including synthetically engineered fondaparinux, heterogeneous LMWH preparations, and UFH. Factor XIII activation is potentiated by fibrin (green dotted arrow), creating a localized positive feedback loop. As detailed in Section 2.1, the pharmacological profiles of anticoagulants differ based on their molecular structure and mechanism of action, with distinctive inhibitory patterns against specific coagulation factors.

The differential activity between unfractionated heparin (UFH) and LMWHs stems from polysaccharide chain lengths. UFH shows equipotent inhibition of factor Xa and thrombin (1:1 ratio) due to extended saccharide sequences exceeding 18 units, enabling efficient AT–thrombin bridging. In contrast, LMWH demonstrates preferential factor Xa inhibition (2–4:1 anti-Xa:anti-IIa ratio), as only one-third of its molecules have sufficient length for AT–thrombin complex formation. The vitamin K antagonist warfarin modulates factors II, VII, IX, and X synthesis, while direct factor Xa inhibitors (apixaban, edoxaban, rivaroxaban, betrixaban) act through small-molecule binding to the active site of factor Xa. Dabigatran and parenteral agents argatroban, hirudin, and lepirudin act through direct thrombin inhibition. The final phase involves fibrinogen undergoing thrombin-dependent proteolysis to generate fibrin monomers, which undergo factor-XIIIa-mediated covalent cross-linking (Figure 2).

Recent structural investigations have revealed additional complexity in heparin’s biological activities. The molecule’s conformational flexibility, particularly in the iduronic acid residues, enables interaction with diverse protein targets beyond the coagulation cascade. These interactions depend on specific sulfation patterns and oligosaccharide sequence arrangements, which influence binding affinity and biological responses. Understanding these structure–function relationships has proven crucial for developing targeted therapeutics with optimized activity profiles.

Multiple regulatory checkpoints exist through AT complexes, including synthetically engineered fondaparinux, heterogeneous LMWH preparations, and UFH. Factor XIII activation is potentiated by fibrin (green dotted arrow), creating a localized positive feedback loop.

## 3. Heparin Derivatives: Types and Production

The development of heparin derivatives represents a significant advancement in anticoagulant therapy, with LMWHs emerging as particularly important therapeutic agents. These derivatives are produced through various depolymerization processes of unfractionated heparin, yielding compounds with distinct molecular and therapeutic characteristics.

### 3.1. LMWH Production Methods and Characteristics

LMWH production involves the controlled fragmentation of unfractionated heparin through specific depolymerization protocols, including chemical hydrolysis, enzymatic degradation, and selective ultrafiltration. These processes yield heterogeneous mixtures of oligosaccharides with molecular weights typically ranging between 3000 and 6500 daltons [16]. While these fragments retain UFH’s fundamental sugar composition, their reduced chain length profoundly alters both pharmacokinetic and pharmacodynamic properties [16,17]. Current European Pharmacopoeia standards define LMWHs as sulfated glycosaminoglycan salts with weight-average molecular weights below 8000 Da and at least 60% of the total mass comprising fragments under this threshold. Most commercial preparations contain oligosaccharides averaging 4000–8000 Da, corresponding to chains of 6–12 disaccharide units—substantially shorter than their UFH precursors. These structural modifications enhance key clinical parameters, including selective factor Xa inhibition, predictable dose–response relationships, and improved subcutaneous bioavailability [18].

The synthesis of LMWHs preserves the heparin backbone structure, while modifications to the terminal ends of the oligosaccharide chains occur through chemical or enzymatic depolymerization, producing unique chemical configurations at both the reducing and non-reducing ends. These distinct terminal structures vary across different LMWH types and can influence binding affinities, particularly for AT, due to the selective cleavage of highly sulfated versus undersulfated regions of the heparin chain [19]. The structural variations that arise from different depolymerization methods impact LMWHs’ pharmacological behavior, underscoring the need for thorough structural analysis and quality control to ensure consistency across LMWH preparations.

Regulatory-approved LMWHs available in various markets include dalteparin (Fragmin^®^), enoxaparin (Lovenox^®^), nadroparin (Fraxodi^®^), tinzaparin (Innohep^®^), parnaparin (Fluxum^®^), bemiparin (Hibor^®^, Zibor^®^, Badyket^®^), ardeparin (Normiflo^®^), reviparin (Clivarin^®^), and certoparin (Sandoparin^®^, Embolex^®^). Table 1 presents an overview of the structural and production techniques, including molecular weights, anti-FXa ratios, and degrees of sulfation for these LMWHs. Among these, dalteparin, enoxaparin, and tinzaparin are particularly prominent, with average molecular weights between 4.0 and 7.0 kDa. The anti-FXa to anti-FIIa ratios of LMWHs typically range from 1.5 to 3.5, which reflects their pharmacodynamic profiles and clinical use for preventing and managing thromboembolic disorders [20].

Various depolymerization techniques produce distinct molecular and anticoagulant profiles suited for specific therapeutic applications (Table 2). These methods are commonly categorized as follows:

1. Deaminative Cleavage with Nitrous Acid (HONO):

Used to produce LMWHs like dalteparin and nadroparin, this method selectively cleaves N-sulfated glucosamine residues, creating anhydro-D-mannose at the reducing end [22,23]. The resulting LMWHs have preserved disaccharide units, like L-iduronosyl-2-O-sulfate and N-sulfo-D-glucosamine-6-O-sulfate, but show around 10% reduction in N-sulfated glucosamine content compared to peroxide-cleaved LMWHs [24]. These nitrous-acid-prepared LMWHs display lower molecular weights and narrower size distributions than peroxide-processed LMWHs, with anti-Xa to anti-IIa ratios ranging from 1.5 to >10 [31]. The deaminative method may produce N-nitroso byproducts, necessitating further purification [32].

2. Alkaline β-Elimination:

This technique, applied in the production of enoxaparin and bemiparin, cleaves glycosidic bonds, yielding LMWHs with high anti-Xa activity and minimal anti-IIa effects, which enhances anticoagulant activity with reduced bleeding risks [25,26]. Enoxaparin and bemiparin show distinct anti-Xa ratios and lower mean molecular weights. For instance, bemiparin has the lowest molecular weight and highest anti-Xa ratio among LMWHs, which suits it well for thrombosis prevention with minimal bleeding risks [26].

3. Enzymatic Depolymerization:

Tinzaparin is produced by enzymatic depolymerization of UFH with heparinase, allowing for selective cleavage under mild conditions and resulting in LMWHs with favorable pharmacokinetics for DVT prevention and treatment [27,28]. Enzymatic methods maintain bioactivity while providing controlled fragment sizes and anti-Xa to anti-IIa ratios between 1.5 and 2.5 [28]. Tinzaparin’s structure retains many of heparin’s core characteristics with fewer processing artifacts [33].

4. Oxidative Depolymerization:

Using agents such as hydrogen peroxide and metal ions (e.g., Cu^2^⁺, Fe^2^⁺), oxidative depolymerization involves a radical chain mechanism targeting unsulfated uronic acid bonds, which can modify or disrupt the antithrombin binding pentasaccharide sequence due to its essential unsulfated glucuronic acid unit. This results in products with altered anticoagulant profiles compared to the parent heparin [29,30]. This process, which may use ultrasonic assistance, yields LMWHs with high anti-Xa ratios and reduced polydispersity [34]. These LMWHs, featuring fewer nonsulfated uronic acid residues, maintain significant anticoagulant properties even with lower molecular weights [30].

### 3.2. Manufacturing Processes and Scale-Up

The pharmaceutical production of heparin faces substantial economic and logistical challenges. The 2008 contamination crisis involving oversulfated chondroitin sulfate necessitated implementation of enhanced analytical platforms and diversification of source materials, significantly increasing production costs [35]. The predominant reliance on porcine intestinal mucosa creates supply chain vulnerabilities and sustainability concerns, while strict regulatory requirements and quality control measures add considerable manufacturing expenses. Rising global demand, coupled with limited raw material availability, has driven efforts to explore alternative sources, including the reintroduction of bovine-sourced heparin and investigation of marine organisms [36].

These economic pressures particularly impact the development of specialized heparin derivatives, where complex manufacturing processes and rigorous safety testing requirements further escalate production costs. Complex extraction and purification protocols significantly affect yield, while stringent quality control requirements implemented after the 2008 crisis have necessitated enhanced analytical testing methods. The challenges of sustainable sourcing persist alongside difficulties in scaling up synthetic alternatives, creating ongoing pressure on production capabilities.

Initial isolation of raw heparin involves tissue extraction from animal sources, primarily porcine intestinal mucosa. The complex purification sequence includes proteolytic digestion, ion-exchange chromatography, and multiple precipitation steps to remove protein contaminants and other glycosaminoglycans. Scaling these processes for industrial production requires specialized equipment and carefully controlled conditions to maintain consistent product quality. The depolymerization methods for LMWH production present additional manufacturing challenges, including precise control of reaction conditions, effective neutralization of chemical reagents, and comprehensive purification to eliminate potential toxic byproducts.

Biological assays remain essential for confirming the functional activity of heparin products, with anti-Xa and anti-IIa chromogenic assays serving as the primary methods for potency determination. Additional specialized testing includes heparanase inhibition assays, selectin binding evaluations, and growth factor interaction analyses for products intended for applications beyond anticoagulation. Regulatory compliance requires comprehensive validation of analytical methods and establishment of appropriate reference standards to ensure batch-to-batch consistency in commercial production.

### 3.3. Desulfation Patterns in LMWHs: Anti-Inflammatory and Anticancer Properties

LMWHs demonstrate biological activities extending beyond their established anticoagulant effects, encompassing anti-inflammatory and anticancer properties. Modulation of these activities correlates with specific sulfation patterns within LMWH molecules, a characteristic directly influenced by the depolymerization methodologies employed in their synthesis [37]. Structural modifications at reducing and non-reducing termini, while resultant from specific degradation processes, exhibit complex structure–activity relationships requiring further mechanistic elucidation.

The hypothesis that LMWHs manifest distinct biological signatures corresponding to unique sulfation configurations necessitates rigorous experimental validation. Beyond the well-characterized antithrombin binding pentasaccharide sequence, empirical evidence supporting discrete sulfation configurations within heparin molecules remains limited [37,38,39]. The complexity of establishing definitive structure–activity relationships is evident in the comparative analysis presented in Table 2, which examines pathophysiological activities associated with LMWHs synthesized via diverse methodologies.

Current literature demonstrates a limited number of comprehensive comparative analyses examining the anticancer properties of enoxaparin versus the antimetastatic effects of tinzaparin. Additionally, systematic assessment of relative heparanase inhibition efficacy among different LMWHs remains inadequately characterized, despite this enzyme’s critical role in tumor progression and metastasis. Experimental evidence indicates that tinzaparin, generated through enzymatic depolymerization, demonstrates significant anti-inflammatory properties and marked antimetastatic effects, particularly regarding heparanase inhibition [40]. However, the molecular mechanisms underlying these pharmacological effects require further elucidation through systematic investigation.

### 3.4. Advanced Applications and Future Directions

Through decades of clinical investigation, LMWHs have proven indispensable for managing thrombotic risk, particularly in challenging scenarios involving malignancy or major surgery [41]. Traditional UFH dominated early anticoagulation protocols, but comprehensive data supporting LMWHs’ superior pharmacodynamic profile have transformed clinical practice [42]. The distinctive molecular structure of LMWHs—characterized by reduced size—yields enhanced bioavailability and more predictable anticoagulant responses through diminished binding to both endothelial surfaces and plasma proteins [43].

A pivotal advantage of LMWH therapy emerges in its markedly reduced propensity to trigger heparin-induced thrombocytopenia compared to conventional UFH regimens [44]. Research across diverse surgical populations has documented substantially lower rates of both HIT occurrence and subsequent thrombotic complications with LMWH prophylaxis [45,46]. This protective effect appears especially pronounced in postoperative settings, where HIT risk traditionally peaks. Mechanistically, the reduced formation of platelet factor 4-heparin complexes directly attenuates the immunological cascade responsible for HIT pathogenesis [46]. These compelling safety advantages have established LMWHs as preferred agents across numerous high-risk clinical scenarios, from orthopedic procedures to trauma care [47].

Beyond standard thromboprophylaxis, heparins serve crucial roles in specialized clinical contexts, including cardiopulmonary bypass, hemodialysis, and acute coronary syndrome management [48]. While bleeding risks and HIT potential remain important considerations in specific patient subgroups [49], heparins continue to demonstrate unmatched value in preventing thromboembolic events. Their impact is particularly evident in surgical populations, where they significantly reduce pulmonary-embolism-associated mortality [50]. This robust evidence base, combined with decades of clinical experience, reinforces heparins’ central position in modern anticoagulation protocols.

Future developments in heparin production technology are exploring synthetic and semi-synthetic pathways to reduce dependence on animal-derived materials. Chemoenzymatic approaches utilizing bacterial fermentation of heparosan followed by enzymatic modification represent a promising alternative production route. These methods could potentially enhance product purity, reduce batch-to-batch variability, and mitigate contamination risks associated with traditional extraction processes. Additionally, development of site-specifically modified heparins with enhanced pharmacological properties continues to advance, with targeted chemoenzymatic synthesis enabling precise control over sulfation patterns and molecular weight distribution.

## 4. Cancer and Heparin: Clinical Strategies for Thrombosis Prevention and Potential Therapeutic Benefits

### 4.1. Cancer-Associated Thrombosis Management

LMWHs have demonstrated complex interactions with cancer progression and survival outcomes. While early studies suggested potential survival benefits in cancer patients treated with LMWHs, subsequent meta-analyses have yielded inconsistent results across diverse oncology populations [51,52,53,54]. However, LMWHs show clear efficacy in preventing venous thromboembolism (VTE), particularly in high-risk populations such as patients with advanced pancreatic cancer [55]. The compounds have also demonstrated favorable safety profiles in specific clinical scenarios, including thromboprophylaxis during gastric cancer surgeries [56].

Clinical research examining LMWH use in cancer patients has produced mixed outcomes regarding overall survival benefits. While systematic reviews consistently demonstrate VTE risk reduction compared to traditional anticoagulants [57,58], the impact on patient survival appears limited primarily to early-stage malignancies, though these findings have not been consistently reproduced across different cancer types [53,54]. Safety analyses from meta-analyses reveal that, while LMWHs may increase minor bleeding events compared to placebo, they show comparable rates of major hemorrhage to standard anticoagulation approaches [57,59]. Notably, heparin-induced thrombocytopenia occurs significantly less frequently with LMWHs than with unfractionated heparin based on data from large cohort studies [59,60].

### 4.2. DOACs in Cancer-Associated Thrombosis

Recent clinical investigations have substantially advanced our understanding of DOAC efficacy in cancer-associated thrombosis. The SELECT-D, ADAM-VTE, and Caravaggio trials validate rivaroxaban, apixaban, and edoxaban as viable alternatives to LMWH [61,62]. These landmark studies provide robust evidence supporting the use of DOACs in cancer patients, though important considerations remain.

DOACs represent a paradigm shift in thrombosis management, offering distinct advantages over conventional agents [63]. These synthetic molecules selectively target specific coagulation factors, primarily thrombin or factor Xa. The therapeutic arsenal includes dabigatran (thrombin inhibitor) and factor Xa antagonists rivaroxaban, apixaban, and edoxaban [64]. Their clinical utility stems from predictable pharmacokinetics and elimination of routine monitoring requirements [65].

While demonstrating comparable efficacy in preventing recurrent VTE, these studies revealed differential bleeding risks across cancer subtypes, particularly in gastrointestinal and urological malignancies [66]. The oral administration route offers practical advantages for many patients, potentially improving treatment adherence and quality of life. However, individualized risk stratification remains paramount, considering factors such as cancer type, stage, concurrent medications, and patient preferences when selecting optimal anticoagulation strategies.

The pharmacological profiles of DOACs demonstrate important variations that influence their clinical application. Dosing strategies vary among agents: apixaban requires twice-daily administration, while rivaroxaban and edoxaban maintain efficacy with daily dosing. Rivaroxaban’s absorption profile mandates food co-administration for higher doses (15–20 mg), whereas apixaban and edoxaban demonstrate food-independent absorption. This distinction in absorption characteristics has important implications for patient compliance and therapeutic effectiveness.

The development of specific reversal protocols, including idarucizumab for dabigatran and andexanet alfa for factor Xa inhibitors, has enhanced the safety profile of these agents by providing options for rapid anticoagulation reversal in emergency situations.

Pharmacogenetic testing may enable more personalized approaches to anticoagulation therapy, potentially improving both efficacy and safety outcomes. The ongoing development of novel anticoagulants focuses on achieving greater target specificity while minimizing bleeding risks. Investigation of factors XI and XII inhibitors represents a promising direction, potentially offering anticoagulation without significantly compromising hemostasis.

These therapeutic advances provide a foundation for understanding heparin’s expanded role in cancer treatment, which extends beyond traditional anticoagulation mechanisms to encompass direct antineoplastic effects through various molecular pathways.

### 4.3. Safety Considerations and Long-Term Effects

Dosing and Mortality Risk: The dose-dependent effects of extended heparin therapy in cancer patients require precise management. Higher therapeutic doses correlate directly with increased bleeding risk [67], with mortality and hospitalization rates rising significantly with excessive anticoagulation [68]. Prophylactic doses maintain better tolerability profiles while preserving therapeutic efficacy.

Age-Related Considerations: In elderly patients, altered drug metabolism leads to heightened bleeding risks [69], with those over 75 years experiencing nearly twice the rate of hemorrhagic complications. Different tumor types present unique risk considerations—brain malignancies require particularly careful anticoagulation management given the devastating potential of intracranial bleeding. Research has shown notably higher bleeding rates in patients with gastrointestinal and genitourinary cancers receiving therapeutic anticoagulation [70].

Organ Function Impact: Prolonged heparin treatment significantly impacts bone metabolism, though LMWHs demonstrate reduced effects on bone density compared to UFH [71]. Impaired renal function necessitates dose modifications due to reduced clearance and potential drug accumulation [72]. Liver dysfunction alters coagulation factor synthesis and metabolism, mandating close monitoring of clotting parameters [73]. These physiological changes become increasingly relevant during extended treatment periods.

Monitoring Requirements: Systematic monitoring must include blood count assessment, coagulation parameters, and organ function evaluation [74]. Early detection of bleeding manifestations, thrombotic complications, and treatment adherence remains essential [75]. Multiple medication regimens in cancer treatment demand particular attention to drug interactions, especially with antiplatelet agents and chemotherapy protocols.

Advanced Disease Considerations: Advanced disease states introduce additional complexities regarding both safety and efficacy. Careful monitoring becomes essential when managing patients with compromised organ function or those receiving concurrent chemotherapy, as these factors can significantly alter LMWH pharmacokinetics and bleeding risk. These findings highlight the importance of individualized risk assessment and careful patient selection.

Long-Term Management: Regular assessment and laboratory monitoring enable rapid intervention for emerging complications [43,76]. Individualized risk assessment combined with systematic safety monitoring optimizes therapeutic outcomes. Current protocols for extended therapy continue to evolve [41], particularly regarding patient-specific risk factors and monitoring intervals. This becomes especially critical when dealing with long-term anticoagulation in cancer patients who may require extended treatment periods.

### 4.4. Clinical Trials and Therapeutic Applications of Heparin Derivatives in Cancer Treatment

#### 4.4.1. Pivotal Clinical Trials by Cancer Type

Lung Cancer Trials: The FRAGMATIC trial (NCT00519805) assessed dalteparin addition to standard treatment in 2202 lung cancer patients. The administration of dalteparin resulted in a reduction of VTE incidence from 9.7% to 5.5%. However, this reduction did not translate into improved overall survival or metastasis-free survival rates [77]. The trial also explored other critical outcomes, including patients’ quality of life and the cost-effectiveness of dalteparin as part of the treatment protocol [78]. A subsequent meta-analysis focused on anticoagulation strategies in lung cancer patients lacking standard indications for anticoagulation therapy reported improved survival rates at 1 and 2 years, particularly among those with non-advanced stages and small cell lung cancer [79]. Additionally, some studies have indicated that dalteparin may enhance survival in non-metastatic cancer patients with VTE [80].

Brain Cancer Trials: Investigation of dalteparin’s therapeutic potential in NCT00028678 revealed no enhancement of overall survival when administered concurrently with radiation therapy in glioblastoma patients, though it significantly reduced thromboembolic complications [81]. High-grade glioma patients exhibit particular vulnerability to VTE, with elevated risk following surgical procedures including biopsy and subtotal resection [82]. While prophylactic LMWH administration appears to effectively mitigate VTE risk without substantially elevating intracranial hemorrhage incidence [83], hemorrhagic complications remain a critical consideration [84]. Subsequent investigations into LMWH integration with standard treatment protocols have yielded conflicting outcomes—several studies suggest modest improvements in progression-free and overall survival metrics, while others demonstrate no appreciable survival advantage [84,85,86]. Emerging preclinical evidence points to inherent antineoplastic properties of heparin and its derivatives, warranting investigation of N-acetyl-cysteine heparin mimetics as potential therapeutic agents [87].

Pancreatic Cancer Trials: The clinical investigation NCT00031837 focused on assessing dalteparin’s effects on patient well-being when treating inoperable or metastatic pancreatic cancer. Traditional gemcitabine therapy, though well-established, has seen marked improvements through recent therapeutic combinations. Clinical data indicate that incorporating dalteparin into gemcitabine protocols led to noteworthy reductions in VTE occurrence while extending patient survival intervals [88].

NCT00462852 investigated the therapeutic potential of LMWH–gemcitabine combinations in advanced pancreatic cancer (APC). Both dalteparin and tinzaparin demonstrate significant VTE risk reduction in APC patients [88,89]. Tinzaparin incorporation correlates with enhanced progression-free survival, suggesting potential antineoplastic effects. Meta-analytic findings confirm LMWH efficacy in VTE prevention without significantly increasing major bleeding events [90]. While definitive survival benefits remain under investigation, current evidence supports the safety and feasibility of LMWH–chemotherapy combinations [91]. Mechanistic investigations suggest LMWH modulation of PAR-1 and KRAS signaling pathways, both implicated in pancreatic cancer progression [92].

Gastrointestinal (GI) and Genitourinary (GU) Cancer Trials: Phase 3 MAGNOLIA research (NCT05171075) currently examines how abelacimab, which targets factor XI, compares to standard dalteparin treatment for managing VTE in patients with GI and GU cancers. The development of cancer-linked VTE introduces substantial complications that worsen patient outcomes and increase treatment complexity, while placing additional strain on healthcare resources [93]. While LMWHs have historically outperformed vitamin K antagonists, daily injection requirements present considerable patient burden [94]. Direct oral anticoagulants (DOACs) offer streamlined administration protocols [95]. Abelacimab’s investigation centers on its potential hemostasis-sparing properties, particularly relevant for cancer patients with elevated bleeding risk [96]. Contemporary clinical protocols emphasize patient-specific thromboprophylaxis strategies [97], with ongoing research focused on optimizing VTE management across diverse oncological contexts [98].

The GASTRANOX investigation (NCT00718354) assesses enoxaparin’s clinical utility and safety parameters in patients with unresectable or advanced gastric/gastroesophageal carcinomas. The protocol combines daily enoxaparin administration with standard chemotherapeutic agents—epirubicin, cisplatin, and capecitabine—over a six-month treatment window. Primary endpoints encompass overall survival metrics and symptomatic thromboembolic event frequencies. The study cohort comprises patients aged 18–75 with stage III/IV gastric adenocarcinoma, aiming to elucidate LMWH’s role in survival optimization and thrombotic risk management within this patient population.

Hepatic Cancer Trials: NCT06153394 examines thromboelastography (TEG^®^) applications for hypercoagulability detection in hepatic cancer surgical interventions. The trial utilizes continuous coagulation parameter tracking for early identification of thrombotic tendencies, which enables precise prophylactic intervention planning. Through extended anticoagulation measures tailored to individual risk factors, the investigators seek to reduce postoperative thrombotic events. TEG^®^ methodology potentially enables more granular risk stratification for VTE prevention, particularly relevant given the heightened thrombotic susceptibility during perioperative phases of hepatic cancer treatment.

#### 4.4.2. Methodological Limitations Across Clinical Trials

While the clinical trials examining heparin derivatives in oncology provide valuable insights, a critical assessment reveals significant methodological limitations that warrant careful consideration. The FRAGMATIC trial offers a particularly illuminating example of these complexities. Although the study demonstrated that dalteparin effectively reduces the incidence of blood clots in lung cancer patients, the trial team concluded that the medication’s benefits are counterbalanced by an increased bleeding risk. Critically, the trial found no improvement in overall patient survival, challenging the broader therapeutic assumptions about LMWHs in cancer treatment [78]. The researchers ultimately recommended that future investigations should focus on identifying specific high-risk subgroups of lung cancer patients who might benefit most from anticoagulation therapy. More broadly, trials like FRAGMATIC, MAGNOLIA, and GASTRANOX exhibit substantial limitations in statistical power and generalizability. The inherent variability across different cancer types, treatment protocols, and patient cohorts introduces significant confounding factors that are not comprehensively addressed in existing research. These methodological constraints underscore the necessity for more nuanced, targeted clinical investigations that can definitively establish the therapeutic potential of heparin derivatives across diverse oncological applications.

#### 4.4.3. Clinical Considerations for Different Cancer Types

Different tumor types present unique risk considerations for LMWH therapy. Brain malignancies require particularly careful anticoagulation management given the devastating potential of intracranial bleeding. Research has shown notably higher bleeding rates in patients with gastrointestinal and genitourinary cancers receiving therapeutic anticoagulation [99,100]. Advanced disease states introduce additional complexities regarding both safety and efficacy. Careful monitoring becomes essential when managing patients with compromised organ function or those receiving concurrent chemotherapy, as these factors can significantly alter LMWH pharmacokinetics and bleeding risk. These findings highlight the importance of individualized risk assessment and careful patient selection when considering LMWH therapy in cancer patients.

### 4.5. Direct Antineoplastic Applications

Current research demonstrates the expanding therapeutic potential of heparin derivatives in oncology, extending beyond traditional anticoagulation mechanisms. While initial studies suggested potential survival benefits in cancer patients treated with LMWHs, subsequent meta-analyses have yielded inconsistent results across diverse oncology populations [51,52,53,54]. However, LMWHs have shown clear efficacy in preventing VTE, particularly in high-risk populations such as patients with advanced pancreatic cancer [55].

The compounds have demonstrated favorable safety profiles in specific clinical scenarios, including thromboprophylaxis during gastric cancer surgeries [56]. Clinical research examining LMWH use in cancer patients has produced mixed outcomes regarding overall survival benefits. While systematic reviews consistently demonstrate VTE risk reduction compared to traditional anticoagulants [57,58], the impact on patient survival appears limited primarily to early-stage malignancies, though these findings have not been consistently reproduced across different cancer types [53,54].

Combination strategies with conventional cancer therapies have shown promise. The integration of dalteparin with gemcitabine in pancreatic cancer demonstrated improved outcomes [88], while tinzaparin’s addition to standard protocols has suggested enhanced progression-free survival [89]. These findings indicate potential synergistic effects when LMWHs are combined with conventional chemotherapy regimens.

### 4.6. Implementation Considerations

The findings from FRAGMATIC, MAGNOLIA, GASTRANOX, and other pivotal trials have substantially influenced contemporary oncology practices, establishing evidence-based protocols for anticoagulation in cancer patients. Current guidelines strongly favor LMWH over vitamin K antagonists for the initial 6-month treatment of cancer-associated thrombosis, supported by Level 1A evidence. This preference stems from demonstrated superior efficacy and the predictable pharmacokinetic profile of LMWH compounds. Implementation requires systematic risk stratification, typically utilizing validated tools such as the Khorana score, which enables identification of high-risk patients who may benefit most from prophylactic anticoagulation [101].

Clinical protocols have evolved to address specific cancer subtypes, with particular attention to pancreatic and lung malignancies. In pancreatic cancer, prophylactic dalteparin has shown utility in locally advanced and metastatic disease, though careful patient selection remains crucial. The FRAGMATIC trial’s findings have informed a risk-stratified approach to thromboprophylaxis in lung cancer, balancing therapeutic benefit against bleeding risks. These protocols typically incorporate regular monitoring of platelet counts and renal function, with dose adjustments based on body weight and creatinine clearance [102].

Integration of anticoagulation into cancer care demands careful coordination between oncology and anticoagulation services. Treatment timing must account for chemotherapy cycles, surgical interventions, and potential drug interactions. Standardized protocols now exist for managing breakthrough thrombosis and adjusting therapy during invasive procedures. Safety monitoring encompasses regular assessment of bleeding risk, surveillance for heparin-induced thrombocytopenia, and careful attention to renal function, particularly in patients receiving nephrotoxic chemotherapy agents [103].

The translation of clinical trial findings into practice has highlighted several implementation challenges. These include the need for consistent risk assessment, standardized monitoring protocols, and clear communication channels between different specialty services. Healthcare systems have developed various strategies to address these challenges, including electronic health record integration of risk assessment tools, standardized order sets, and dedicated anticoagulation services for cancer patients. Regular audit of outcomes has enabled continuous refinement of these protocols, leading to improved patient care and reduced complications [104].

## 5. Molecular Mechanisms of Heparin’s Antitumor Effects

Clinical data indicate hemostatic irregularities in 15–25% of oncology patients [105]. Coagulation cascade activation affects both systemic and local tumor dynamics, influencing initiation, progression, and metastatic spread [106]. Systemic manifestations include DVT and metastasis, while local effects produce fibrin and plasma protein accumulation within tumoral spaces [107]. Such fibrin matrices influence tumor architecture, immune cell migration, neovascularization, and stromal maturation [108]. The resultant elevation in interstitial pressure from protein and fibrin buildup impedes chemotherapeutic penetration [109].

### 5.1. Heparan Sulfate Structure and Function in Normal and Malignant States

As a widely distributed glycosaminoglycan, heparan sulfate (HS) mediates numerous biological processes through protein interactions [110,111]. These HS-binding proteins span multiple categories—from growth factors and chemokines to enzymes and matrix components [112,113]. The molecular makeup of HS reveals a complex polysaccharide with alternating glucuronic/iduronic acid and glucosamine units, featuring heavily sulfated NS domains interspersed between less modified NA segments [114]. This distinctive arrangement emerges through enzyme-driven modifications, beginning with N-deacetylase/N-sulfotransferases (NDSTs) [115]. Both sulfation patterns and domain organization vary by tissue type, directly impacting protein binding and biological effects [116]. The spacing and length of NS domains, combined with flexible unsulfated regions, enable HS to serve multiple biological functions [117].

HS and its proteoglycan forms (HSPGs) regulate both normal cell functions and cancer development through protein binding that influences cell signaling, adhesion, and differentiation pathways [111]. The diverse structures of HS chains and specific protein motifs control these interactions [118]. In cancer tissue, disrupted HS and HSPG patterns emerge, affecting tumor growth, angiogenesis, metastasis, and immune evasion [119]. Cancer cells show altered HS patterns and increased heparanase production, particularly affecting growth factor pathways like FGF, VEGF, and PDGF [112].

### 5.2. Therapeutic Targeting of HS–Protein Interactions

Targeting HS–protein interactions has yielded multiple therapeutic strategies, including structurally modified heparins, small-molecule inhibitors, and monoclonal antibodies [120]. HS mimetics show remarkable potential by interfering with growth factor signaling while augmenting standard treatment protocols [121]. These compounds inhibit tumor growth through multiple mechanisms—disrupting neovascularization, limiting cancer cell proliferation, and reducing metastasis [122,123]. Research shows they effectively block both heparanase and sulfatase activity [124]. The development pipeline includes several promising approaches, from neutralizing antibodies to peptide-based mimetics, at various clinical testing stages [125]. Advanced analytical methods in HS sequencing and structural characterization guide the rational design of HS-inspired therapeutic molecules [126]. This targeting of HS–protein interactions represents an innovative strategy to enhance antineoplastic efficacy and address therapeutic resistance.

### 5.3. Heparanase Inhibition and Sulfation Patterns in Anticancer Activity

Heparanase functions as a key endoglycosidase in heparan sulfate degradation, playing central roles in metastatic spread and neovascularization [127]. Tumoral heparanase upregulation correlates with enhanced metastatic potential, increased vascular density, and reduced patient survival intervals [128]. The enzyme works through dual mechanisms—direct HS cleavage and non-enzymatic signaling effects—to facilitate cellular invasion, growth factor mobilization, and vascular development [129,130]. Experimental data demonstrate that targeted heparanase suppression effectively limits both tumor advancement and metastatic spread [131]. Beyond cancer, heparanase influences inflammation, autoimmune conditions, and diabetic nephropathy [132].

Sulfation patterns play a crucial role in modulating heparin’s anticancer properties, particularly in relation to heparanase inhibition. Highly sulfated heparin enhances antimetastatic and antiangiogenic activities through dual mechanisms: improved heparanase inhibition and more effective blocking of P-selectin-mediated tumor cell adhesion [133,134]. Analysis of heparin derivatives reveals dual importance of N-sulfate and O-sulfate groups in heparanase inhibition, with N-sulfation requirements of minimally one group per disaccharide unit. Heparin compounds exhibit variable heparanase inhibitory capacities based on sulfation patterns, N-acetylation status, and glycol-split modifications [134].

Structural requirements for optimal heparanase inhibition include specific N-sulfate/N-acetyl distribution, strategic O-sulfation, and minimum oligosaccharide length of 16 units [127]. Although 2-O and 3-O sulfation influence overall activity, they are not essential for specific anticancer effects, as evidenced by 2,3-O-desulfated heparin maintaining its heparanase inhibition capabilities. This important finding suggests the potential for developing modified heparins that preserve their therapeutic properties while carrying reduced bleeding risk [134]. These structurally modified heparins demonstrate concurrent antiangiogenic and antimetastatic properties while exhibiting reduced anticoagulant activity [135].

The relationship between specific sulfation patterns and anticancer activity extends beyond heparanase inhibition. Sulfation patterns directly influence heparin’s interactions with growth factors, cytokines, and adhesion molecules involved in tumor progression. The desulfating enzyme SULF1 has been identified as a tumor suppressor, further emphasizing the importance of sulfation balance in cancer biology [136]. Different structural requirements exist for various anticancer activities, which enables the strategic design of heparin derivatives with optimized therapeutic profiles [137].

In comparative analyses of different heparin derivatives, distinct relationships emerge between sulfation patterns and biological activities. LMWHs prepared through different depolymerization methods, as detailed previously in Table 2, demonstrate varying capacities for heparanase inhibition. Enzymatically depolymerized heparins like tinzaparin show particularly potent heparanase inhibition (IC_50_ ~0.5–1 µg/mL), while those prepared via oxidative methods typically exhibit weaker inhibitory effects (IC_50_ ~8–15 µg/mL for ardeparin) [134,138]. This variability correlates with specific sulfation patterns maintained or altered during the depolymerization process.

The ability of heparin derivatives to disrupt tumor cell adhesion and metastasis is also significantly influenced by sulfation patterns. P-selectin inhibition, which prevents platelet–tumor cell aggregation and promotes monocyte interaction [139], depends on specific sulfation arrangements that are independent of anticoagulant effects [140]. Additional mechanisms include endothelial P-selectin targeting, which relies on particular sulfation configurations [141]. These patterns can be selectively modified through chemical techniques described in Section 6.2, including targeted desulfation, N-acetylation, and glycol splitting, to optimize anticancer properties while minimizing hemorrhagic risks.

The extensive research on heparanase inhibition and sulfation patterns has revealed important structure–activity relationships that guide the development of specialized anticancer heparin derivatives. While complete mechanistic understanding requires further investigation, the current evidence provides a clear foundation for designing optimized compounds. These findings emphasize the critical importance of specific sulfation patterns in determining heparin’s diverse biological activities, extending well beyond its conventional anticoagulant functions to encompass sophisticated modulation of tumor progression and metastasis through multiple molecular pathways.

### 5.4. Interference with Tumor Cell Adhesion and Metastasis

Heparin modulates cellular proliferation through protein-kinase-C-dependent pathways, proto-oncogene suppression, cell cycle regulation, ERK pathway inhibition, and apoptotic induction [142]. Its antimetastatic properties involve multiple mechanisms: adhesion molecule inhibition, growth factor pathway modification, tissue factor pathway inhibitor release, and chemokine signaling disruption [143].

Selectin-mediated interactions between tumor cells, platelets, and endothelium represent key targets. P-selectin inhibition prevents platelet–tumor cell aggregation, promoting monocyte interaction [144], independent of anticoagulant effects [145]. Additional mechanisms include endothelial P-selectin targeting [146], heparanase inhibition, and growth factor modulation [147]. Heparin suppresses proto-oncogenes and vascular smooth muscle progression [148,149], though complete mechanistic understanding requires further investigation [150].

### 5.5. Angiogenesis Modulation and CXCR4/CXCR7 Signaling

Heparin’s therapeutic potential extends significantly beyond anticoagulation, demonstrating complex interactions with tumor vasculature, growth patterns, and metastatic processes [43,151]. The compound exerts direct antineoplastic effects through multiple mechanisms, including modulation of cellular adhesion pathways, regulation of growth factor activity, and immunological modification [152,153]. Research has consistently demonstrated heparin’s capacity to inhibit various aspects of tumor progression, including cellular proliferation, adhesion mechanisms, invasive potential, and metastatic dissemination [141,154].

**Figure 3:**
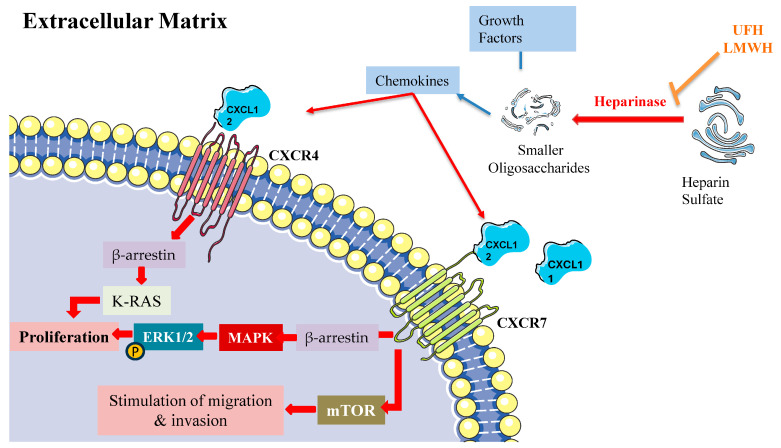
β-arrestin-dependent signaling pathways of CXCR4 and CXCR7 in the extracellular matrix. The figure depicts the β-arrestin-dependent signaling mechanisms of CXCR4 and CXCR7 chemokine receptors in the extracellular matrix. Heparinase cleaves heparin sulfate into smaller oligosaccharides (UFH, LMWH), leading to the release of chemokines. CXCL12 activates CXCR4, while both CXCL11 and CXCL12 act as cognate agonists for CXCR7 (ACKR3). Upon activation, CXCR4 recruits β-arrestin, which triggers K-RAS activation and subsequent phosphorylation of ERK1/2 via the MAPK cascade to promote cell proliferation. CXCR7 activation also leads to β-arrestin recruitment, activating two distinct pathways: (1) the MAPK/ERK1/2 cascade resulting in proliferation, and (2) mTOR signaling promoting cell migration and invasion. This signaling network illustrates the β-arrestin-mediated cellular responses in the extracellular matrix microenvironment.

LMWH’s antitumor effects operate through several key pathways, notably through heparanase inhibition, modulation of P- and L-selectin activity, suppression of angiogenic processes, and interference with CXCL12/CXCR4 signaling (Figure 3). Within this complex network, the chemokine receptors CXCR4 and CXCR7 emerge as critical regulators of cancer progression and metastasis, primarily through β-arrestin-dependent signaling cascades. These receptors frequently demonstrate co-expression patterns in cancer cells, with their heterodimeric interactions leading to sustained β-arrestin recruitment and enhanced cellular migration capabilities.

The molecular mechanisms underlying CXCR4/CXCR7 signaling reveal sophisticated regulatory patterns. Both receptors activate ERK1/2 through β-arrestin-2-mediated pathways, while CXCR7 additionally activates mTOR signaling [155]. The system demonstrates complex competitive dynamics, with CXCR7 competing for β-arrestin-2 recruitment against CXCR4, thereby modulating CXCL12-mediated responses [156]. CXCR4 activation triggers MEK1/2 and Akt phosphorylation cascades, promoting invasive cellular behavior [157]. Furthermore, the coordination between β-arrestin-1 and STAM1 plays a crucial role in regulating focal adhesion kinase autophosphorylation and CXCR4-dependent chemotactic responses [158].

While CXCL12 is the cognate agonist of CXCR4, both CXCL11 and CXCL12 function as cognate agonists of CXCR7 (ACKR3). CXCR4’s activation by its cognate ligand CXCL12 initiates multiple downstream signaling events, prominently including MAPK cascade activation leading to ERK1/2 phosphorylation and subsequent cellular proliferation [159]. This process involves complex interplay between several pathways, including Ras/Raf signaling, Src kinase activation, and Rho/ROCK pathway engagement. The signaling cascade encompasses K-Ras activation and β-arrestin-2 recruitment, both essential for ERK1/2 phosphorylation [155]. Additionally, CXCR4 activation stimulates ARF1, a small G protein that interacts with Raf1 to enhance MAPK activation, particularly in prostate cancer cells [160]. This signaling network has established CXCR4 as a promising therapeutic target across various pathological conditions, including cancer and inflammatory disorders.

### 5.6. Immunomodulatory Effects

LMWHs enhance immunotherapeutic protocols through increased lymphocytic infiltration, particularly cytotoxic CD8^+^ T cells. This enhancement stems partly from vascular normalization effects, reducing immunosuppression within the tumor microenvironment [161]. Studies document improved survival metrics in LMWH-treated cancer patients [38]. The complexity of heparin’s effects necessitates precise dosing strategies [76]. Careful antiangiogenic modulation can optimize vascular function, potentially amplifying immunotherapy response [162].

Mechanistic studies reveal heparin’s interference with immune checkpoint pathways and enhancement of antigen presentation. The compound’s ability to normalize tumor vasculature may facilitate improved immune cell trafficking and function within the tumor microenvironment. These effects, combined with heparin’s established anti-inflammatory properties, suggest potential synergistic benefits when combined with modern immunotherapy approaches, though optimal dosing and timing strategies require further investigation.

## 6. Non-Anticoagulant (NAC) Heparin Derivatives: Development and Applications in Cancer Therapy

Chemical modifications of LMWH molecules occur through distinct depolymerization processes, yielding compounds with specific pharmacological and structural characteristics adapted for multiple therapeutic applications [163]. Within cancer therapeutics, N-acetylated-cysteine (NAC) heparin derivatives show particular efficacy in metastasis prevention [164]. Strategic modifications, including N-acetylated heparin (NACH) and its desulfated variant (D-NACH), maintain essential protein binding interactions while demonstrating negligible anticoagulant effects [165]. Selective chemical alterations—including de-O-sulfation, de-N-sulfation, and re-N-acetylation processes—enable precise optimization of NAC heparins for targeted therapeutic outcomes with reduced side effect profiles [166,167]. These compounds exhibit potent inhibitory effects on heparanase activity, a crucial enzyme in metastatic progression [135]. Integration of NAC heparins into nanoparticle delivery platforms further augments their therapeutic potential in oncological applications [168].

The anticoagulant properties of conventional heparins present challenges for their use in cancer therapy due to bleeding risks [169]. Researchers have thus explored NAC variants and heparin–drug conjugates to maintain anticancer effects while reducing hemorrhagic complications [164,170]. LMWHs and ULMEHs are viable options for preventing VTE in cancer patients, highlighting the need for ongoing research to refine heparin-based treatments [41]. Studies reveal that NAC heparin derivatives can effectively reduce bleeding risks while retaining antitumor efficacy, offering a promising approach to safer cancer treatment [76].

Novel heparin derivatives demonstrate expanded therapeutic potential. Clinical evaluation of necuparanib, a heparin mimetic, revealed antitumor activity in pancreatic cancer, reducing proliferation and metastasis [171]. Trials confirmed its compatibility with standard treatment protocols [172,173]. Recent developments include acetylated low-anticoagulant LMWH (ALMWH), synthesized via sodium periodate oxidation and borohydride reduction, demonstrating concentration-dependent antiproliferative effects in MDA-MB-231 breast cancer cells (IC_50_: 22.16 µM, 48 h exposure) [174].

### 6.1. Determinants of Heparin’s Anticoagulant Activity

The anticoagulant activity of sulfated polysaccharides, including heparin, is influenced by their specific structural features, notably sulfation patterns and types of glycosylation. Key features include 2-sulfation in α-L-sulfated galactans, 2,4-di-sulfation in α-L-fucopyranosyl units, and a high degree of 4-sulfation alongside 2-sulfation in dermatan sulfate [175]. Furthermore, the 3-O-sulfation of glucosamine is crucial to heparan sulfate’s biological functions [176]. Anticoagulant potency increases with specific sulfate group positioning, with certain di-sulfated units exhibiting higher activity [177]. Different structural aspects also influence the mechanism of action, with some sulfated fucans acting directly on thrombin, while others rely on co-factors such as antithrombin or heparin co-factor II [178].

### 6.2. Producing NAC Heparin Derivatives: Chemical Modification Methods

NAC heparins can be synthesized through various chemical modifications to reduce anticoagulant activity while preserving other biological functions [165]. Other important techniques include selective O-desulfation [179], N-acetylation, and complete desulfation [180]. These modifications can produce heparin derivatives with diverse structures and reduced anticoagulant properties [181]. While nitrous acid treatment is valuable for structural analysis of heparin, it is primarily used as an analytical tool rather than a production method for NAC heparins [182]. The resulting modified heparins retain various pharmacological activities, such as heparanase inhibition, antiangiogenic effects, and antitumor properties [183] (Table 3).

#### 6.2.1. Periodate Oxidation and Glycol Splitting in Heparin Derivatives

Periodate oxidation of UFH and LMWHs selectively cleaves unsulfated glucuronic acid residues, resulting in derivatives with reduced anticoagulant activity but preserved other biological functions [165,184]. The methodology targets adjacent diol groups within unsulfated glucuronic acid domains, generating ring-opened configurations that preserve growth factor binding capabilities and anti-inflammatory activity. Structural modification of the antithrombin binding region substantially diminishes heparin’s anticoagulant effects. Glycol-split heparin synthesis proceeds through dual chemical transformations: initial periodate-mediated oxidation of non-sulfated uronic acid moieties, followed by borohydride-induced reduction [184]. The oxidative phase selectively cleaves vicinal diols in unsulfated glucuronic acid regions to form transient aldehyde intermediates [182]. Subsequent reduction converts these aldehydes to corresponding alcohols, introducing conformational flexibility into the molecular framework [184]. This sequential transformation effectively reduces anticoagulant activity through specific disruption of AT recognition sites [185]. The glycol-splitting process enhances heparanase inhibition properties, targeting a key enzyme in metastatic progression [135]. These modified NAC heparin derivatives maintain critical biological functions, including anti-inflammatory and antineoplastic activities, suggesting significant therapeutic potential [165].

#### 6.2.2. Alkaline Treatment and Chemical Modifications in Heparin Derivatives

Further structural modifications can be achieved through alkaline treatment and acid hydrolysis, yielding various oligosaccharide fragments [186]. Additionally, certain terminal amino sugar residues in LMWHs have been found to be susceptible to periodate oxidation [187]. The periodate oxidation process can be refined by combining chemical modifications and enzymatic depolymerization, with the order of treatments significantly impacting the final structure and activity of heparin derivatives [181]. In the traditional glycol splitting of heparin, a two-step approach, unsulfated glucuronic acid residues undergo periodate oxidation, while glucosamine residues are resistant to glycol cleavage regardless of their sulfation status, being protected by their N-sulfation or N-acetylation pattern [184]. However, introducing an alkylation step between oxidation and reduction fundamentally alters this selectivity [188]. The alkylation process removes these protective sulfate groups, effectively “unmasking” previously protected sites and leading to widespread modification across all regions during the subsequent reduction step [183]. Notably, alkaline conditions promote desulfation, particularly at the 2-O and 3-O positions of iduronic acid in heparin [183]. This chemical behavior has been leveraged to study structure–function relationships. For instance, studies with 2,3-O-desulfated heparin have revealed that these sulfate positions are not essential for heparanase inhibition, provided that other sulfate groups are retained [135], suggesting functional redundancy or the importance of other structural features in this specific biological activity.

#### 6.2.3. N-Acetylation and N-Deacetylation: Modulating Heparin’s Anticoagulant Properties

N-deacetylation and N-desulfation modifications substantially alter heparin derivatives’ anticoagulant characteristics and physiological distribution [189]. These acetylation processes serve dual roles in synthetic modification and natural biosynthetic pathways. Chemical N-acetylation utilizes acetic anhydride under precise conditions to prevent unwanted O-acetylation events [190], a methodology particularly relevant for bioengineered heparin development and biological property optimization. Within natural biosynthetic pathways, microsomal N-deacetylase enzymes catalyze selective acetyl group removal from N-acetylglucosamine residues [191]. The distribution and extent of these deacetylation events direct subsequent structural modifications, including N-sulfation patterns and uronic acid epimerization processes.

Bioengineered heparin production requires precise control over the proportion and sequence of N-sulfated versus N-acetylated glucosamine residues to achieve structural and functional equivalence with pharmaceutical-grade products [192]. The specific distribution pattern of N-sulfated and N-acetylated glucosamine residues along the polysaccharide chains directly influences protein binding interactions, anticoagulant potency, and other therapeutic effects. Strategic manipulation of these N-substitution patterns enables development of novel heparin-based therapeutics with customized biological profiles and enhanced safety parameters.

#### 6.2.4. Optimizing NAC Heparins: Selective O-Desulfation

Selective O-desulfation of heparin produces NAC heparin derivatives that retain various pharmacological activities while minimizing bleeding risks. These derivatives can be obtained by removing 2-O and 3-O sulfates [179] or through partial N-desulfation [37]. They exhibit reduced anticoagulant properties but maintain effectiveness in inhibiting neutrophil proteases, complement activation, and cell proliferation [179]. NAC heparins can also preserve bone morphogenetic protein-2 bioactivity [193] and inhibit cancer progression by interfering with heparanase activity and selectin-mediated interactions [194]. Specific desulfation techniques, such as using N-methylpyrrolidinone–water mixtures, allow for regioselective O-6-desulfation [195]. These modified heparins demonstrate anti-inflammatory properties, including inhibition of complement activation and leukocyte adhesion, without significant anticoagulant effects [196].

#### 6.2.5. Complete Desulfation Methods for Heparin

Complete desulfation of heparin requires carefully controlled chemical conditions depending on the targeted sulfate groups. Solvolytic desulfation using DMSO with 5–10% water and pyridine predominantly affects 6-O-sulfate groups [195]. For selective 6-O-desulfation, N-methyl-N-(trimethylsilyl)trifluoroacetamide can be employed under specific reaction conditions [197]. Total desulfation can be achieved through methanolic HCl treatment under strictly anhydrous conditions, though this requires precise temperature control and extended reaction times [190]. For selective 2-O-desulfation, alkaline treatment with NaOH provides consistent results when carefully monitoring pH and temperature [198]. The selection of desulfation method significantly impacts the characteristics of the resulting NAC heparin derivative. Reaction parameters including temperature range, pH, solvent composition, and duration must be precisely controlled to achieve the desired degree of desulfation while preserving the heparin backbone structure. More extensive desulfation generally requires stronger reaction conditions, increasing the risk of unwanted structural modifications.

### 6.3. NAC Heparins: Anticancer Mechanisms

NAC heparins exhibit considerable anticancer potential through mechanisms that extend beyond their anticoagulant properties. These include the inhibition of tumor cell migration, modulation of immune responses, and disruption of growth factor activity [194]. Targeted elimination or deactivation of antithrombin binding domains enables NAC heparins to retain metastasis-inhibiting functions while reducing hemorrhagic complications [194]. Studies demonstrate that NAC heparin derivatives within the 8–10 kDa range effectively suppress metastatic progression in experimental models, showing no interference with primary tumor dynamics or coagulation parameters [199,200]. These compounds act through multiple pathways: disrupting selectin-mediated cellular adhesion, suppressing heparanase function, and modifying angiogenic processes [141,164]. NAC heparins modulate critical tumor dissemination pathways by altering cancer cell interactions with platelets, immune cells, and vascular endothelium [164]. Parallel investigations of LMWH compounds reveal comparable metastasis inhibition, correlating with enhanced survival metrics in preclinical models [201,202]. These findings suggest particular therapeutic potential for NAC heparins in highly metastatic malignancies, including melanoma, warranting expanded clinical evaluation. Additionally, NAC heparin derivatives have been found to inhibit selectins, angiogenesis, and the CXCL12–CXCR4 axis, all of which are vital in cancer metastasis [134]. They also disrupt P-selectin-mediated interactions between tumor cells and platelets, which may contribute to reduced metastasis [144]. Furthermore, NAC heparins show promise in inhibiting galectin-3, a known promoter of metastasis, highlighting their potential as safe and effective anticancer agents [203].

The structural requirements for different anticancer activities of NAC heparins vary, which allows for the design of specific heparin derivatives [137]. Sulfation patterns are particularly important in mediating heparin’s anticancer effects, with SULF1, a heparan-sulfate-desulfating enzyme, exhibiting tumor suppressor properties [136]. While the potential of heparins as anticancer agents is evident, their complex effects on cancer progression necessitate further research to optimize their therapeutic applications.

The translation of NAC heparin derivatives from preclinical research to clinical oncological applications presents formidable challenges. Despite promising laboratory findings, significant scientific and regulatory hurdles remain unresolved. These compounds theoretically offer anticancer effects without the bleeding risks associated with traditional heparins. Yet this potential advantage demands rigorous validation through comprehensive trials. Higher therapeutic dosing without proportional hemorrhagic complications sounds appealing but requires systematic confirmation.

Much remains unknown about their precise mechanisms of action. Researchers must elucidate exactly how these compounds inhibit metastasis at the molecular level. Long-term safety profiles across multiple organ systems need thorough characterization. Can these derivatives demonstrate consistent efficacy across various cancer types? The current evidence base suffers from methodological inconsistencies and limited follow-up data.

Future investigations must employ standardized compounds and validated biomarkers. Only methodologically robust studies with clinically relevant endpoints will determine whether NAC heparins deserve a place in our therapeutic arsenal. The path from promising laboratory curiosity to approved clinical intervention remains long and uncertain. Yet the potential benefits justify continued research into these intriguing compounds.

## 7. Innovations in Heparin-Based Therapeutic Development

Chemoenzymatic synthesis advances enable production of unmodified heparin oligosaccharides, expanding therapeutic applications while minimizing hemorrhagic complications. Dociparstat sodium (DSTAT), a 2-O, 3-O desulfated heparin variant, represents a significant advancement with reduced anticoagulant effects while preserving anti-inflammatory activity [204]. Developed through Cantex Pharmaceuticals before Chimerix acquisition, DSTAT operates via multiple mechanisms: complement cascade inhibition, adhesion molecule binding, and disruption of leukocyte–RAGE interactions [196]. Preclinical studies demonstrate its efficacy in reducing both myocardial reperfusion injury and pulmonary metastasis [196,204]. The compound’s anti-inflammatory properties stem primarily from P- and L-selectin inhibition, with 6-O-sulfate groups playing crucial structural roles [205]. DSTAT effectively suppresses neutrophil elastase-mediated inflammation in cystic fibrosis models [206].

Chemically modified heparin derivatives inhibit heparanase, an enzyme linked to tumor progression, angiogenesis, and metastasis [207]. These derivatives successfully prevent heparanase-mediated degradation of heparan sulfate [137] and may inhibit galectin-3 binding, potentially obstructing metastatic processes [203].

The production of DSTAT involves selective desulfation, treating unfractionated heparin with acid at pH 1.5–2.0 and 50–55 °C for 18–24 h [208]. This process specifically targets 2-O and 3-O sulfate groups, reducing anticoagulant activity while preserving other pharmacological properties [179,209]. The selectivity of depolymerization methods significantly affects sulfation patterns. The synthesis requires a strong acidic environment, elevated temperatures, and prolonged reaction times, specifically cleaving O-sulfate ester bonds while preserving the heparin backbone structure. This mechanistic approach enables nitrous acid depolymerization to reduce molecular weight while maintaining critical sulfation patterns.

Clinical investigations reveal DSTAT’s therapeutic promise in acute myeloid leukemia (AML) management. The compound interferes with leukemia stem cell homing through CXCL12/CXCR4 pathway modulation, disrupting bone marrow protective niche interactions [210]. Integration with conventional chemotherapy yields improved complete remission rates and accelerated platelet recovery [211,212]. DSTAT’s efficacy stems from multiple mechanisms, including platelet factor 4 binding and HMGB1 inhibition [210]. Its ability to disrupt leukemia cell–bone marrow microenvironment interactions may help overcome chemotherapy resistance [213]. Combining DSTAT with targeted therapies could enhance outcomes by addressing various resistance mechanisms in AML [214].

To build upon the clinical trial results described in this review, we propose critical preclinical and translational research priorities to advance the development of LMWHs and NACHs as potential anticancer agents. Structure–activity relationship studies represent a primary research imperative. Comprehensive investigations are needed to define optimal sulfation patterns that maximize antitumor efficacy while minimizing anticoagulant effects, with particular focus on non-anticoagulant heparins (NACHs). This necessitates developing sophisticated methods for precisely controlling sulfation during LMWH and NACH synthesis, coupled with extensive profiling of sulfation pattern variations across diverse in vitro and in vivo cancer models.

Comparative studies emerge as a crucial next step. Rigorous head-to-head evaluations of multiple LMWHs across preclinical tumor models representing varied cancer types and molecular subtypes are essential. Such comprehensive screening will facilitate identification of lead candidates demonstrating the broadest and most potent antitumor activity, enabling strategic prioritization for future clinical trials.

Rational combination strategies warrant systematic exploration, leveraging the established mechanisms of action of LMWHs. Preclinical investigations should methodically assess these agents in combination with targeted chemotherapeutic drugs, molecular therapies, and immunotherapeutic approaches where preliminary evidence suggests potential synergistic interactions.

Finally, in-depth investigations into the multifaceted impacts of LMWHs and NACHs on the tumor microenvironment, immune response dynamics, and drug resistance mechanisms could unveil novel therapeutic intervention strategies. These studies are critical for comprehensively understanding the complex biological interactions of these promising compounds.

## 8. Conclusions and Future Directions

Heparin derivatives, including LMWHs and NAC heparins, demonstrate therapeutic versatility in oncology through multiple mechanisms. The targeted modification of heparin’s sulfation patterns provides precise control over biological activities, as summarized in Table 4. Notably, derivatives with high N- and 6-O-sulfation effectively inhibit heparanase activity, while specific sulfation arrangements disrupt both CXCL12/CXCR4 signaling cascades and selectin-mediated metastatic processes.

Clinical trials including FRAGMATIC, MAGNOLIA, and GASTRANOX have validated the safety and efficacy of these compounds in managing cancer-associated thrombosis, while providing preliminary evidence of direct antineoplastic effects in specific cancer subtypes. The emerging role of structurally modified heparins, exemplified by dociparstat sodium, demonstrates how targeted alterations can enhance therapeutic specificity while minimizing hemorrhagic complications.

Significant challenges persist in optimizing these compounds for clinical use. Manufacturing complexity demands improved synthetic strategies for controlling sulfation patterns while minimizing off-target effects. Additionally, comprehensive studies across diverse cancer types must identify responsive patient populations and guide clinical applications. The combination of heparin derivatives with existing anticancer agents, including chemotherapeutics and immunotherapies, represents a promising avenue for enhancing treatment outcomes.

While initial safety data support the therapeutic potential of heparin derivatives, the field urgently needs robust long-term safety studies. Current evidence gaps include comprehensive assessments of chronic toxicities, cumulative organ effects, and potential interactions with extended cancer treatments. Systematic evaluation of bone health, immunological responses, and cardiovascular impacts during prolonged therapy remains particularly critical. The development of standardized monitoring protocols must precede widespread clinical implementation.

NAC heparins emerge as particularly promising alternatives, offering targeted anticancer effects while minimizing bleeding risks associated with traditional heparin derivatives. Their selective modification allows retention of key therapeutic properties while reducing anticoagulant activity, potentially enabling higher dosing and expanded clinical applications in oncology.

The continuing evolution of heparin derivatives in oncology marks a significant advancement in cancer treatment strategies. By addressing these structural, mechanistic, and clinical considerations, researchers can overcome current limitations and fully realize the therapeutic potential of these versatile compounds. Through continued interdisciplinary collaboration and systematic investigation, these compounds may substantially improve outcomes across multiple cancer types, particularly in settings where current therapeutic options remain limited.

## Figures and Tables

**Table 1:** A comparative summary of commercial LMWH and ULMWH, detailing key molecular properties, manufacturing patents, and anticoagulant characteristics. Each entry includes the International Nonproprietary Name (INN) of the heparin derivative, alongside its common commercial brand names and the primary manufacturer. The “Original Patent” column specifies the main patent reference, which defines the intellectual property protection for each compound or process. The “Preparation Method” column highlights the technique used to produce each derivative, as indicated in the primary patent’s Claim 1; claim types specify whether the patent protection covers a product, process, or product-by-process. The “MW (Da)” column provides the molecular weight range in Daltons, reflecting the molecular size profile of each compound. Structural details for the non-reducing end (NRE) and reducing end (RE) are included to represent key molecular features resulting from the depolymerization process. Finally, each entry provides the anti-Xa activity ratio, anti-Xa and anti-IIa activities (IU/mg), and the degree of sulfation per saccharide unit (average number of sulfate (SO_3_) groups attached to the sugar residues). In UFH, the average degree of sulfation is typically around 2.5. Note that sevuparin’s anti-Xa and anti-IIa activities are unusually low (<10 IU/mg for both) compared to typical LMWH values, reflecting its design to retain antiadhesive properties while significantly reducing anticoagulant activity [21,22,23,24,25,26,27,28,29,30].

LMWH/ULMWH (INN)	Brand Name(s)	Manufacturer	Original Patent	Preparation Method (Claim Type/Claim 1)	MW (Da)	NRE	RE	Anti-Xa:Anti-IIa Ratio	Anti-Xa Activity (IU/mg)	Anti-IIa Activity (IU/mg)	Degree of Sulfation
Dalteparin sodium	Fragmin, Boxol, FR 860	Pfizer/Kabi/Pharmacia-Upjohn	EP0014184A2	Compound/Heparin fragments, 14–18 sugar units with L-iduronosyl-2-O-sulfate-N-sulpho-D-glucosamine-6-O-sulfate	5600–6400	2-O-sulfo-α-L-idopyranosuronic acid	6-O-sulfo-2,5-anhydro-D-mannitol	1.9–3.2:1	110–210	35–100	2.0–2.5
Enoxaparin sodium	Lovenox, Clexane	Sanofi-Aventis/Rhone-Poulenc	US4990502	Product/Composition of low-molecular-weight heparins and pharmaceutically acceptable salts	3500–5500	2-O-sulfo-4-enepyranosuronic acid	2-N-sulfated-D-glucosamine, 1,6-anhydro ring	3.3–5.3:1	100–210	20–35	~2.0
Tinzaparin sodium	Innohep, Logiparin	LEO Pharma/Novo Nordisk	EP0244235	Process/Production of LMW-heparin by enzymatic depolymerization	5500–7500	2-O-sulfo-4-enepyranosuronic acid	2-N,6-O-disulfo-D-glucosamine	1.5–2.5:1	70–120	45–50	2.66
Nadroparin calcium	Fraxodi, CY-216	Sanofi-Winthrop	DE2944792	Compound/Mucopolysaccharide fraction from heparin-based material	4200–5500	2-O-sulfo-α-L-idopyranosuronic acid	6-O-sulfo-2,5-anhydro-D-mannitol	2.5–4.0:1	95–130	27–37	2.0–2.5
Bemiparin sodium	Zibor, Hibor, Badyket	Rovi	EP0293539	Process/Depolymerization of heparin with MW 10,000–20,000 Da	3000–4200	2-O-sulfo-4-enepyranosuronic acid	2-N,6-O-disulfo-D-glucosamine	8.0:1	80–100	10–12.5	~2
Sevuparin	N/A	Modus Therapeutics AB/Dilafor AB	WO2002072799A1	Compound/Heparin derivative with specified structural formula	6500–9500	2-N,6-O-disulfo-D-glucosamine	Glucosamine bound to a remnant	1.5:1	<10	<10	2.4
Parnaparin sodium	Fluxum, Minidalton	Alfa Wassermann SpA	EP0294099B1	Process/Oxidative depolymerization with Cu^2^⁺ and H_2_O_2_	4500–5000	2-O-sulfo-α-L-idopyranosuronic acid	2-N,6-O-disulfo-D-glucosamine	1.5–3.0:1	75–110	25–30	2.15
Reviparin sodium	Clivarin	Knoll AG/Abbott	EP0467206B1	Compound/Formulation based on heparin, glycosaminoglycan, or heparinoids	3400–4650	2-O-sulfo-α-L-idopyranosuronic acid	6-O-sulfo-2,5-anhydro-D-mannitol	4.2:1	124	29	2.0–2.6
Ardeparin sodium	Normiflo	Wyeth-Ayerst	US5374715A	Process/Production of low molecular weight heparins with high pharmacological properties	2000–15,000	2-O-sulfo-α-L-idopyranosuronic acid	2-N-acetyl-6-O-sulfo-D-glucosamine	1.8:1	95–145	45–75	2.0–2.7
Certoparin sodium	Sandoparin, Alphaparin	Novartis/Sandoz	US4351938	Process/Reacting heparin salt with nitrous acid solution	4200–6200	2-O-sulfo-α-L-idopyranosuronic acid	6-O-sulfo-2,5-anhydro-D-mannitol	1.5–2.5:1	80–120	30–35	2.0–2.5

**Table 2:** Characteristics of LMWHs: Synthesis Methods, Mechanisms, and Clinical Applications. A structured comparison of LMWHs, organized by preparation methods, mechanisms, anti-Xa to anti-IIa ratios, applications, desulfation patterns, and anti-inflammatory or anticancer effects. Each preparation method describes the chemical or enzymatic process used to fragment UFH into LMWH. Commercial LMWHs are listed with examples in parentheses, while the mechanism column details the type of reaction. The anti-Xa to anti-IIa ratio indicates selective inhibition of factor Xa versus factor IIa (thrombin), where higher ratios suggest greater specificity for factor Xa. Applications describe the primary clinical uses. The desulfation status column describes the chemical modifications that occur during the depolymerization process, with specific attention to the types and positions of sulfate groups affected. The final column highlights documented anti-inflammatory and anticancer effects.

Preparation Method	LMWH Examples	Mechanism	Typical Anti-Xa to Anti-IIa Ratio	Applications	Desulfation Status and Location	Anti-Inflammatory and Anticancer Effects
Deaminative Cleavage with Nitrous Acid	Dalteparin (Fragmin), Nadroparin (Fraxodi), Reviparin (Clivarin)	Selective cleavage at glucosamine residues by deaminative reaction	2–4:1	Balanced anticoagulant activity suitable for general anticoagulation	Partial desulfation, mainly at N-sulfated glucosamine residues	Dalteparin: Strong evidence for anticancer effects, approved for cancer-associated thrombosis. Moderate anti-inflammatory properties through P-selectin inhibition. Moderate heparanase inhibition (IC_50_ ~2–5 µg/mL). Nadroparin shows lower heparanase inhibition (IC_50_ ~5–10 µg/mL)
Alkaline β-Elimination	Enoxaparin (Lovenox), Bemiparin (Zibor, Hibor)	Alkaline treatment targeting glycosidic bonds	3.8–8.0:1	High anti-Xa activity with low bleeding risk, ideal for thrombosis prevention	Cleavage occurs via β-elimination at glycosidic bonds. Under alkaline conditions, selective 2-O-desulfation may occur at iduronic acid residues, while N-sulfated glucosamine residues remain largely intact	Enoxaparin: Significant anti-inflammatory effects through NF-κB pathway inhibition. Demonstrated anticancer properties in both clinical and experimental studies. Strong heparanase inhibition (IC_50_ ~1–3 µg/mL). Bemiparin shows particularly strong heparanase inhibition (IC_50_ ~0.5–2 µg/mL)
Enzymatic Depolymerization	Tinzaparin (Innohep)	Specific enzymatic cleavage using heparinase	1.5–2.5:1	Balanced anticoagulant activity suitable for general anticoagulation	Minimal desulfation, primarily at non-reducing ends. Sulfation pattern largely preserved	Tinzaparin: Notable anti-inflammatory effects and strong antimetastatic properties. Strongest heparanase inhibition among LMWHs (IC_50_ ~0.5–1 µg/mL). Most consistent antiheparanase activity across batches. Particularly effective in inhibiting heparanase activity in cancer
Oxidative Depolymerization	Ardeparin (Normiflo), Parnaparin (Fluxum)	Oxidative cleavage with hydrogen peroxide or copper ions	1.5–3.0:1	Balanced anticoagulant activity suitable for general anticoagulation	Random desulfation possible depending on oxidative conditions, often at uronic acid residues	Limited evidence for significant anti-inflammatory or anticancer effects compared to other LMWHs. Weak to moderate heparanase inhibition (Ardeparin IC_50_ ~8–15 µg/mL, Parnaparin IC_50_ ~5–10 µg/mL). More variable heparanase inhibition between batches

**Table 3:** Overview of Chemical Modification Methods and Their Impact on Sulfation Patterns and Biological Properties of NAC Heparin Derivatives. This table provides an overview of various chemical modification methods used to alter the sulfation patterns of heparin derivatives, detailing the specific mechanisms, effects on sulfation status, and biological implications of each method. Each method targets different structural aspects of heparin, leading to distinct sulfation outcomes. Periodate oxidation selectively targets unsulfated glucuronic acid residues, while glycol splitting preserves the original sulfation pattern through oxidation–reduction steps that increase chain flexibility. Selective O-desulfation removes specific sulfate groups (2-O and 3-O) under solvolytic conditions, maintaining anticoagulant activity and enhancing anti-inflammatory effects. N-acetylation and N-deacetylation modify amine sites, influencing potential N-sulfation sites without directly affecting sulfate groups. Complete desulfation, achieved through solvolytic or acidic methods, removes all sulfate groups, drastically altering biological properties. Each approach’s mechanism and impact on sulfate group preservation provide insights into how chemical modifications can be tailored for therapeutic applications.

Method	Sulfation/Desulfation Status	Biological Implications
Periodate Oxidation	Targets vicinal diols in unsulfated glucuronic acid residues under mildly acidic conditions (pH 4–5), producing ring-opened structures. Reduces anticoagulant activity by approximately 85–95% but retains growth factor binding and anti-inflammatory properties. Initial Sulfation: Preserves original sulfation pattern Resulting Sulfation: Minor or no changes in sulfate groups Mechanism: Selectively cleaves unsulfated glucuronic acid regions Sulfate Group Preservation: Nearly complete (>95%)	Maintains some growth factor activity while significantly reducing anticoagulant properties, suitable for therapeutic applications requiring lower anticoagulation.
Glycol Splitting	Maintains complete original sulfation pattern while significantly reducing anticoagulant activity by more than 95%. Initial Sulfation: Full original sulfation Resulting Sulfation: Sulfate distribution remains unchanged Mechanism: Two-step oxidation–reduction preserving sulfation Sulfate Group Preservation: 100% intact Structural Modification: Creates more flexible chain segments without disrupting sulfation	More comprehensive structural modification leads to NAC heparin derivatives, making it suitable for developing anti-inflammatory agents and targeted therapeutic compounds with reduced anticoagulation effects.
Selective O-Desulfation	Uses solvolytic conditions (DMSO/methanol) to remove 2-O and 3-O sulfate groups selectively. Preserves N-sulfation and core structure, reducing anticoagulant activity by 80–85% but enhancing anti-inflammatory and anticancer potential. Initial Sulfation: Full original sulfation Resulting Sulfation: Removes 2-O and 3-O sulfate groups; N-sulfates preserved Mechanism: Targeted O-sulfate removal under solvolytic conditions Sulfate Group Preservation: N-sulfates fully intact, O-sulfates selectively removed	Reduces anticoagulant activity while enhancing anti-inflammatory and anticancer properties, making it useful for therapeutic applications where both effects are desired.
N-Acetylation	Adds acetyl groups to amine sites, modifying biological properties but not sulfate groups. Initial Sulfation: Full original sulfation Resulting Sulfation: No direct change to sulfate groups; acetyl groups added at amine sites Mechanism: Acetylation of free amine positions Sulfate Group Preservation: 100% intact Additional Effect: Blocks potential future N-sulfation sites	Alters biological interactions, potentially affecting downstream signaling and cellular responses.
N-Deacetylation	Removes acetyl groups from N-acetylglucosamine, facilitating future N-sulfation. Initial Sulfation: May have acetylated groups Resulting Sulfation: Removes acetyl groups, generates free amine sites for resulfation Mechanism: Deacetylation of N-acetylglucosamine residues Sulfate Group Preservation: No direct impact on sulfates	Enhances the potential for structural modifications that can influence biological function and interactions.
Complete Desulfation	Removes all sulfate groups, commonly achieved via solvolytic desulfation or methanolic HCl treatment, resulting in non-anticoagulant derivatives. Initial Sulfation: Fully sulfated Resulting Sulfation: Complete sulfate removal, yielding non-sulfated polysaccharides Mechanism: Solvolytic desulfation (DMSO/methanol), methanolic HCl, or pyridine–borane complex Sulfate Group Preservation: 0% (complete removal)	Completely alters biological properties, rendering compounds suitable for applications requiring non-anticoagulant characteristics.

**Table 4:** Comprehensive Applications of Heparin and Heparin Derivatives in Clinical and Research Settings. This table summarizes the various applications of heparin and its derivatives, detailing specific heparin types (UFH, LMWH, NAC heparins, and modified heparins), clinical uses, mechanisms of action, key benefits, and special considerations. Key considerations include monitoring and dosing adjustments for anticoagulant therapy, cancer treatment benefits, anti-inflammatory applications, and novel uses in antiangiogenic and antimetastatic therapies. The research and development category highlights the investigational use of modified heparins, particularly in drug delivery systems and tissue engineering.

Application Category	Heparin Type	Clinical Uses and Mechanisms	Key Benefits	Special Considerations
Anticoagulant Therapy	UFH, LMWH	- Prevention and treatment of VTE, particularly in high-risk patients (e.g., postsurgical, cancer) - Perioperative prophylaxis - Acute coronary syndromes (e.g., myocardial infarction)	- Rapid onset of action (especially UFH) - Reversible with protamine for UFH - Lower bleeding risk with LMWH	- LMWH preferred due to lower risk of HIT - UFH requires regular aPTT monitoring - LMWH may require dose adjustment in renal impairment
Cancer Therapy	LMWH, NAC heparins	- Antitumor activity by inhibiting heparanase and modulating tumor microenvironment - Synergistic enhancement of chemotherapy and immunotherapy efficacy - Prevention of cancer-associated thrombosis, especially in pancreatic and lung cancer	- Inhibition of heparanase enzyme - Reduced metastasis and cancer cell adhesion - Improved survival outcomes in cancer patients	- LMWH effectively reduces VTE risk in cancer patients - NAC heparins minimize anticoagulant effects, reducing bleeding risks while targeting tumors - NAC heparins primarily in experimental use
Anti-Inflammatory Applications	UFH, LMWH	- Management of chronic inflammatory diseases (e.g., asthma, inflammatory bowel disease) - Prevention of thrombotic complications in severe COVID-19 cases - Potential use in other inflammatory conditions	- Reduces inflammatory markers (e.g., cytokines) - Prevents thrombotic events associated with inflammation - Immunomodulatory properties help manage inflammation	- Increased bleeding risk, particularly at high doses - Dose adjustments may be necessary in elderly or those with renal impairment - Long-term use requires monitoring for adverse effects
Antiangiogenic and Antimetastatic	LMWH, NAC heparins	- Inhibition of angiogenesis and metastasis in cancer - Blocking of selectin-mediated tumor cell adhesion, reducing metastatic spread - NAC heparins particularly useful for anticancer without anticoagulation	- Anticancer properties without significant anticoagulation (for NAC heparins) - Effective in reducing tumor angiogenesis and metastatic potential - Fewer systemic side effects	- LMWH has mild anticoagulant effects but is widely used in oncology - NAC heparins primarily in experimental use - Requires further research for optimal dosing and long-term impact
Wound Healing	Topical heparin	- Reduces inflammation at wound sites - Enhances wound healing and tissue repair processes - Reduces risk of excessive scarring and keloid formation	- Local anti-inflammatory effects reduce swelling and promote healing - Accelerates tissue repair - Minimal systemic absorption minimizes bleeding risk	- Limited to topical use due to bleeding risk if absorbed systemically - Specific formulations required for optimal skin absorption
Research and Development	Modified heparins, NAC heparins	- Development of heparin-based drug delivery systems for targeted therapies - Bioengineering applications (e.g., scaffolds for tissue engineering) - Investigational uses in advanced therapeutics (e.g., antiviral therapies)	- Enables targeted delivery to diseased tissues (e.g., tumors) - Reduces off-target effects and systemic side effects - Potential for enhancing therapeutic efficacy in combination therapies	- Primarily in preclinical or early clinical stages - Requires further validation for safety and efficacy - Customization and optimization needed for each therapeutic application

## Data Availability

No original data were generated or analyzed in the preparation of this review article. Data sharing is therefore not applicable.
